# Laparoscopic hernia repair in children: does recreating the open operation improve outcomes? A systematic review

**DOI:** 10.1007/s10029-023-02772-5

**Published:** 2023-03-23

**Authors:** Ayman Goneidy, Christian Verhoef, Nick Lansdale, Robert T. Peters, David J. Wilkinson

**Affiliations:** 1 Department of Paediatric Surgery, Royal Manchester Children’s Hospital, Oxford Road, Manchester, M13 9WL UK; 2 Faculty of Biology Medicine and Health, University of Manchester, Manchester, UK

**Keywords:** Laparoscopic, Hernia repair, Paediatric, Outcomes

## Abstract

**Purpose:**

The use of laparoscopy for paediatric inguinal hernia repairs has increased significantly over the past 2 decades. However, there is significant variation in the reported recurrence rates in the literature, with many studies reporting higher rates than the open operation. This may be explained by the range of different techniques currently included under the term laparoscopic inguinal hernia repair. The purpose of this study is to determine whether dividing the hernia sac before ligation improves surgical outcomes following a paediatric laparoscopic inguinal hernia repair compared to ligation alone.

**Methods:**

A systematic review of the literature was performed following PRISMA guidelines of all studies reporting the outcomes following paediatric laparoscopic inguinal hernia repair where the technique was recorded as laparoscopic suture ligation alone (LS) or laparoscopic sac division and suture ligation (LSDS). Studies were assessed for risk of bias and exclusion criteria included reported follow-up of less than 6 months.

**Results:**

A total of 8518 LS repairs and 6272 LSDS repairs were included in the final analysis. LSDS repair was associated with a significantly lower recurrence rate (odds ratio 0.51, 95% CI 0.36–0.71, *p* = 0.001). There was no significant difference in the rates of testicular ascent or atrophy.

**Conclusion:**

Recreating the open operation by hernia sac division followed by suture ligation significantly reduces the risk of hernia recurrence.

**Supplementary Information:**

The online version contains supplementary material available at 10.1007/s10029-023-02772-5.

## Introduction

Inguinal hernia repair is one of the most frequently performed operations in pediatric practice, with more than 6000 in children under the age of 16 years in England alone in 2019 [1]. The traditional ‘open’ inguinal herniotomy remains the most commonly utilized technique worldwide but since the 1990s there has been growing use of laparoscopy [2–4]. This provides the potential benefits of reduced post-operative pain and quicker recovery, but more importantly has the advantage of visualizing the contralateral deep inguinal ring [5–9]. Yet, it has not achieved the universal acceptance seen with other procedures, such as laparoscopic cholecystectomy which may partly be due to a concern that recurrence rates are higher than those for the open operation thus negating any potential benefits [10, 11].

However, the term laparoscopic hernia repair is used to describe a range of different techniques which may explain the variation in the recurrence rates reported in the literature [11–16]. Despite the heterogeneity in technique, the majority of studies can be broadly divided into two groups: those that replicate the open operation by dividing then ligating the sac versus those that simply ligate it. As with much of the pediatric surgical literature, many of these studies are limited to case series or small, non-randomized comparative studies, making it difficult to draw any firm conclusions.

Therefore, the aim of this study is to perform a systematic review of the literature on pediatric laparoscopic inguinal hernia repairs, to determine whether replication of the open operation (i.e. laparoscopic complete sac dissection and suture (LSDS) cligation) leads to decreased rates of recurrence compared to laparoscopic suture (LS) ligation alone.

## Methods

### Search strategy

This study protocol was designed according to the Preferred Reporting Items for Systematic Review and Meta-analysis (PRISMA) 2020 guidelines [17]. The complete EMBASE and MEDLINE databases were searched from inception until November 2020 using the Medical Subject Headings (MeSH) terms “Inguinal” AND “Hernia” OR “Herniotomy” AND “Laparoscopy” OR “Laparoscopic”. For the population we used the terms “Child” or “Pediatric” or “Paediatric”. Papers selected were restricted to those published in English.

All abstracts identified were then screened by two independent investigators based on our inclusion and exclusion criteria (Table 1).

**Table 1:** Inclusion and exclusion criteria

Inclusion criteria	Exclusion criteria
• Documented outcomes following Laparoscopic indirect inguinal hernia repair • Documented used study techniques: Laparoscopic circumferential dissection and suture (**LCDS**) or Laparoscopic suture (**LS**) • Study population < 18 yrs	• Open inguinal hernia repairs or other abdominal hernias (Direct, femoral, umbilical, epigastric, etc.) • Studies with < 10 patients • Failure to report recurrence rates • Non-English language studies

Although in the initial selection phase all papers reporting outcomes from laparoscopic intracorporeal sutured ring closure were included, for detailed analysis only those papers in which the technique was described as either LSDS or LS and the results were clearly attributable to the individual techniques were kept. The technique of LSDS was defined as complete dissection of the hernial sac at the level of the internal ring, followed by suture ligation of the ring. If the internal ring was ligated with a suture, without division of the sac the technique was classified as a LS ligation. Reference lists of identified articles were manually searched to identify additional studies.

The quality of individual studies was assessed independently by two investigators (AG and CV) using the MINORS criteria [18] for all studies apart from randomized controlled studies (RCTs) for which the ROB-2 assessment tool was used [19]. The MINORS score was chosen as a validated method of identifying and assessing non-randomized surgical studies. Similarly, the ROB-2 assessment tool is appropriately validated for randomized controlled studies. In the case of a discrepancy of more than 2 points between investigators, a final decision was made by the senior authors.

### Data extraction

All included studies were analyzed to extract study characteristics (including country, study design and sample size), population characteristics (age, technique) outcomes (recurrence rates, testicular atrophy/ascent) and any reported post-operative complications. Data extraction was performed independently by AG and CV and any discrepancies resolved by the senior author.

### Statistical analysis

Due to the significant heterogeneity and the lack of trials comparing the two techniques formal meta-analysis was not possible. Data are presented as median (interquartile range) unless otherwise stated. Pooled analysis of non-parametric data was performed using Chi-square with Yates correction. Statistical significance was defined as *p* < 0.05.

## Results

**Fig. 1:**
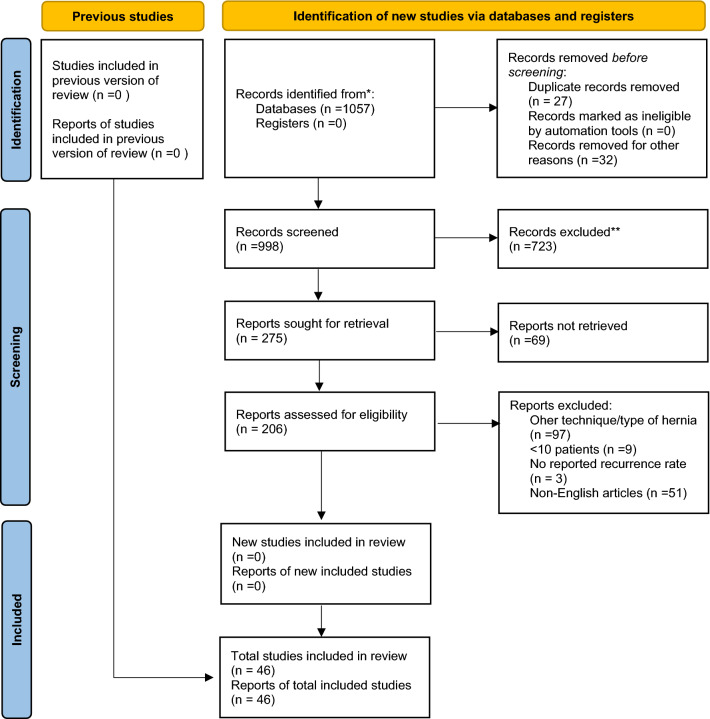
PRISMA flow diagram

The initial search returned 998 abstracts after duplicates were removed (Fig. 1). After full review, only 46 met the full-text inclusion criteria and were selected for final analysis [4, 9–12, 20–62]. These studies included 8 randomized control trials (RCTs), including 2 RCTs comparing LSDS and LS techniques, 3 prospective comparative studies, 5 prospective non-comparative studies, 13 retrospective comparative and 14 retrospective non-comparative which leaves 3 cohort studies (Table 2).

**Table 2:** Studies included

Author	Journal, year	Study design	Technique	Age (Mean)	Number of patients	Follow up (Mean)	Recurrence
Abd-Alrazek M et al	Journal of Pediatric Surgery, 2017	P-RCT	LSDS vs LS	25.3 + /– 7 ms	132	16.8 ms	LSDS 0 Vs 2 LS
Almetaher HA et al	Journal of Pediatric Endoscopic Surgery, 2020	P-CSC	LSDS vs LS	15.3 + /– 19 ms	66	6 ms	LSDS 0 Vs 2 LS
Becmeur F et al	Surgical endoscopy, 2004	R-CS	LSDS	5.9 years	82	6 ms	LSDS 0
Boo YJ et al	Journal of Laparoscopic & Advanced Surgical Techniques, 2012	P-CS	LSDS	40.7 ms	202	12.5 ms	LSDS 0
Borkar NB et al	Journal of Laparoscopic & Advanced Surgical Techniques, 2012	CSC	LSDS & distal sac partial excision vs LSDS	6 ms–12 years (Range)	50	12.4 ms	LSDS 0
Elbatarny AM	Journal of Laparoscopic & Advanced Surgical Techniques, 2020	P-RCT	Lap Sac disconnection vs LSDS	6.7 + /– 7.8 ms	34	7.3 ms	LSDS 0
Esposito C et al	Journal of Laparoscopic & Advanced Surgical Techniques, 2010	R-CS	LSDS	6.7 ms (Median)	315	Minimum 1 year	LSDS 1
Esposito C et al	World Journal of Surgery, 2009	R-CS	LSDS	3.2 years (Median)	315	Minimum 1 year	LSDS 2
Lee SR and Park PJ	Hernia, 2018	R-CS	LSDS	40 + /– 33 ms	51	25 ms	LSDS 0
Lee SR and Park PJ	Surgical endoscopy, 2020	R-CSC	LSDS ± Muscle arch reinforcement	32.2 ms	3386	37.3 ms	LSDS 6 Vs 0 Muscle reinforcement
Montupet P and Esposito C	Surgical endoscopy, 2011	CS	LSDS	54 ms (Median	596	Minimum 6 ms	LSDS 11
Pant N et al	Journal of Indian Association of Pediatric Surgeons, 2014	P-RCT	Laparoscopic Sac dissection vs LSDS	63	57	Not specified	LSDS 1
Shalaby R et al	Journal of Laparoscopic & Advanced Surgical Techniques, 2017	R-CSC	LSDS vs Multiple techniques	24 + /– 25 ms	1284	Not specified	LSDS 0 Vs 8 LS
Tsai YC et al	Surgical endoscopy, 2009	P-CSC	LSDS vs Open	5.4 years	174	21.2 ms	LSDS 1
Van Batavia JP et al	Journal of Pediatric Urology, 2018	R-CS	LSDS	5.9 years (median)	46	Not specified	LSDS 1
Wheeler AA et al	European Journal of Pediatric Surgery, 2011	R-CS	LSDS	36 ms (Median)	26	8 ms	LSDS 0
Lin CD et al	The Journal of Urology, 2011	R-CS	LSDS vs Open	7.2 + /– 4.2 ms	57	22.9 ms	LSDS 0
Esposito C et al	Surgical endoscopy, 2016	R-CS	LSDS	18 ms	1300	28 ms	LSDS 5
Hasanein A et al	The Egyptian Journal of Surgery, 2017	P-RCT	LSDS vs Laparoscopic sac dissection	2 + /– 0.8 years	84	6 ms	LSDS 3
Shehata SM et al	Journal of Laparoscopic & Advanced Surgical Techniques, 2018	R-CSC	LSDS vs Multiple techniques	Phase I 19 ms Vs Phase II 17.6 ms	459	15.5 ms	LSDS 11
Esposito C et al	Pediatric Surgery International, 2012	R-CS	LSDS	Not specified	67	30 ms (Median)	LSDS 3
Ho IG et al	Journal of Pediatric Surgery, 2018	R-CS	LSDS vs Open	2.4 (Median)	465	3.5 years (Median)	LSDS 0
Geiger S et al	Medicine, 2017	R-CS	LS	0–16 years (Range)	119	38 ms (Median)	LS 4
Koivusalo AI et al	Surgical endoscopy, 2007	R-CS	LS vs open	15 ms (Median)	33	26 ms (Median)	LS 1
Koivusalo AI et al	Pediatrics, 2009	P-RCT	LS vs open	6 years (Median)	89	2 years	LS 2
Lee DY et al	Hernia, 2015	R-CS	LS	45.8 ms	98	22.6 ms	LS 0
Lee SR and Choi SB	Hernia, 2016	R-CSC	LS (purse string vs linear suture)	30.5 + /– 29 ms	2223	38.2 & 23.1 ms	LS 11
Li S et al	Journal of Laparoscopic & Advanced Surgical Techniques, 2015	R-CSC	LS	1.5 years (Median)	106	17 ms (Median)	LS 0
Marte A et al	Journal of Laparoscopic & Advanced Surgical Techniques, 2008	R-CSC	LS vs Partial sac dissection + LS	4 years (Median)	224	24 ms	LS 5
Montupet P and Esposito C	Journal of Pediatric Surgery, 1999	CS	LS	4 years	45	> 2 years	LS 2
Pastore V and Bartoli F	Hernia, 2014	R-CS	LS	49 weeks corrected Gestation	30	21 ms	LS 0
Schier F	Journal of Pediatric Surgery, 2006	P-CS	LS	1.6 years (Median)	542	39 ms (Median)	LS 20
Schier F et al	Journal of Pediatric Surgery, 2002	CS	LS	3.2 years	666	40 ms	LS 23
Shalaby R and Desoky A	Pediatric Surgery International, 2002	CS	LS	61.5 + /– 28 ms	150	1 year	LS 0
Shalaby R et al	Journal of Pediatric Surgery, 2014	R-CSC	LS vs Laparoscopic extraperitoneal repair	3 + /– 2 years	874	20 ms	LS 8
Shalaby R et al	Journal of Pediatric Surgery, 2010	P-RCT	LS vs Laparoscopic extraperitoneal repair	61 + /– 28 ms	150	21.9 ms	LS 3
Steven M et al	Journal of Laparoscopic & Advanced Surgical Techniques, 2016	R-CSC	LS vs Open	0.56 years (Median)	254	3.8 years (Median)	LS 3
Turial S et al	Surgical Endoscopy, 2011	R-CS	LS	41 week corrected gestation (Median)	147	26 ms (Median)	LS 4
Turial S et al	Surgical Innovation, 2011	P-CS	LS	2.3 years (Median)	100	42 ms (Median)	LS 2
Ur RF et al	Journal of Medical Sciences, 2019	R-CS	LS	3 years	60	6 ms	LS 4
Walsh CM et al	Surgical Laparoscopic, Endoscopy and Percutaneous Techniques, 2020	R-CS	LS	10.5 weeks (Median)	80	6 ms	LS 1
Yildiz A et al	Pediatric Surgery International, 2012	R-CSC	LS vs Open	6.6 years (Median)	26	8–16 ms	LS 0
Wang F et al	Journal of Laparoscopic & Advanced Surgical Techniques, 2018	R-CSC	LS vs Laparoscopic extraperitoneal repair	46 + /– 30.6 ms	599	20 ms	LS 0
Shou T et al	Hernia, 2018	P-CS	LS	47 ms (Median)	139	13 ms (Median)	LS 0
Kozlov Y and Novozhilov V	Journal of Laparoscopic & Advanced Surgical Techniques, 2015	CS	LS vs Laparoscopic extraperitoneal repair	55.6 Days	260	> 6 months	LS 0
Karadag CA et al	Journal of Experimental and Clinical Medicine, 2016	R-CS	LS vs Multiple techniques	5.6 years	133	48 ms	LS 0

*P* prospective, *R* retrospective, *CS* cohort study, *CSC* cohort study with a control group, *RCT* randomized controlled trial, *LSDS* laparoscopic circumferential sac dissection and sutured repair, *LS* laparoscopic suture repair, *Ms* month, *Yrs* years, *Wks* weeks

**Fig. 2:**
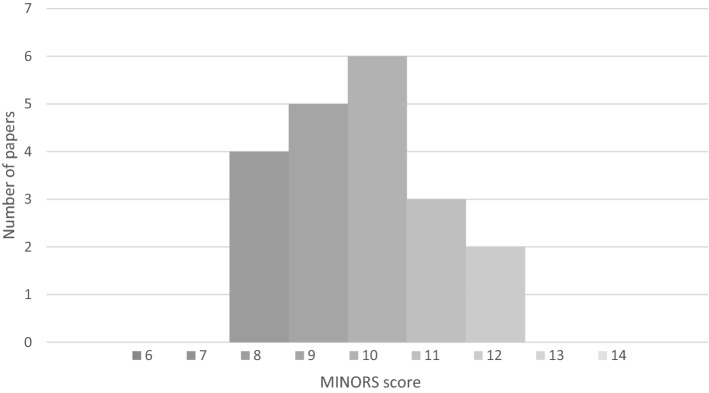
MINORS score for non-comparable studies. Max score 16

**Fig. 3:**
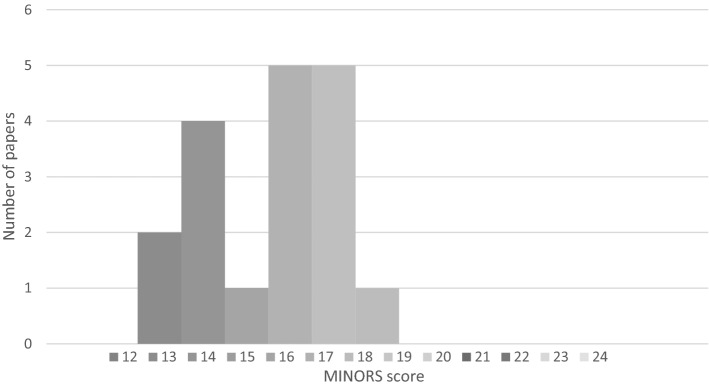
MINORS score for comparable studies. Max score 24

Study quality was reasonable for the comparative studies with a median MINORS score of 16/24 (IQR14-17) and slightly lower for the non-comparative studies with a median score of 10 (IQR 9–10) (Figs. 2–3). A total of 8 non-comparative papers (6 cohort and 3 retrospective cohort studies) were excluded because of poor methodology (taken as a MINORS score of less than 50%). Reason for exclusion was predominantly due to a combination of unmentioned/unclear follow-up period, failure to achieve the targeted follow-up of 95% of the patients, and inclusion of non-consecutive patients.

Following assessment with the RoB-2 tool 5 of 8 RCTs were reported as having a high risk of potential bias. This was often due to the blinding/control methodology used and was not thought to impact on the reporting of recurrence outcomes. Therefore, these studies remained included in the final analysis. Full results of the methodological analysis are included in Supplemental Tables 1, 2, and 3.

In total, these studies included 12555 patients, of which 5520 were in the LSDS group, undergoing 6272 hernia repairs and 7035 in the LS group undergoing 8518 hernia repairs. Study characteristics and patient demographics are reported in Table 1. Median reported follow-up was 22.9 months (15.5–30).

### Recurrence rates

In total, there were 45/6272 (0.7%) recurrences in the LSDS group compared to 120/8518 (1.4%) in the LS group, this gives an odds ratio of 0.51 (95% CI 0.36–0.71), *p* = 0.001. However, as the overall recurrence rate remains low, the absolute risk reduction is 0.8% (95% CI 0.48–1.12%) and a number to treat of 125 (95% CI 89.4–206.6).

### Testicular atrophy/ascent

In the LSDS group, 7 studies (401 patients) reported testicular atrophy as an outcome with no cases of atrophy identified. However, no significant difference was identified between the two groups—2 cases reported in 16 studies (3842 patients) in the LS group (*p* = 1).

With regard to testicular ascent, 8 LSDS studies (2201 patients) reported outcomes for testicular ascent in which 2 cases were identified. Again, no difference was seen between the techniques, with 2 cases in 11 studies (2119 patients) in the LS group (*P* = 0.68). In addition, 6 patients required a later orchidopexy for an undescended testicle identified but not treated at the time of the original hernia repair.

## Discussion

The potential benefits of utilizing a laparoscopic approach for the management of pediatric inguinal hernias, such as to treat metachronous hernias, has prompted many surgeons to develop techniques that provide this benefit without increasing morbidity [5–9]. As such, there are a large number of papers reporting outcomes under the label of a ‘laparoscopic hernia repair.’ However, the umbrella term ‘laparoscopic inguinal hernia repair’ is used to described a wide range of approaches, including sutureless, LS and LSDS repairs [8, 9, 63]. This may explain the significant variation in complication rates reported in the literature [4, 9–12, 20–62] and makes subsequent comparison of studies difficult. This study is the first systematic review to compare two strict definitions to determine whether recreating the open operation—performing a herniotomy before ligating the sac (LSDS) is superior to ligating the sac alone (LS).

The quality of the current literature remains mixed, with large numbers of small studies with inconsistent outcome reporting. Many studies do not provide enough detail of the actual technique and had to be discarded with many more reporting outcomes within weeks of the operation and therefore at high risk of missing recurrences. Furthermore, additional but important outcomes, such as testicular atrophy and ascent are rarely reported.

Despite strict inclusion criteria, the risk of bias in most studies was moderate, this was predominantly due to the lack of comparative groups and small study size. Further potential limitations include the failure to separate study populations out into different risk groups, such as premature infants. However, the size of the combined groups should minimize the effect of these subgroups in the overall analysis.

Despite these issues, we identified a significant reduction in the risk of recurrence associated with the LSDS repair; however, it must be said that the overall recurrence rate remains low for both techniques. This difference may be explained by the reliance of the LS technique on a single suture. If that suture fails, or as is the case in some studies resorbs, then the open hernial sac is still present and the hernia recurs. However, in the LSDS group performing a herniotomy prior to closure provides a second layer of protection.

There are some who suggest that cutting the sac may increase the risk of vas or vessel injury [64] and there is certainly an increased technical challenge in safely dissecting the sac in small infants. These outcomes are poorly reported by many of the included studies and we cannot comment on whether there is a significant learning curve to achieve good results, as it was outside the study parameters. However, we did not identify any increased risk of testicular atrophy associated with sac dissection and in the authors’ experience [65], we have not found this to be an issue whether the procedure is performed by an experienced surgeon or surgeon in training.

The laparoscopic approach has been found to be associated with lower rates of testicular ascent compared to the open repair; however, the reason why remains unclear [66]. We had postulated that division of the sac would further prevent the testis from becoming tethered to the sutured internal ring and therefore being at risk of subsequent ascent, i.e. that the LSDS repair would be associated with a lower rate of ascent. However, we were unable to identify a significant difference between the groups. Again, thorough analysis was limited by the lack of long-term active follow-up in the papers to specifically address this issue.

This study was limited to intraperitoneal LS repairs only, but there has been increased interest in laparoscopically assisted extraperitoneal repairs. The suggested benefit of the laparoscopic assisted extraperitoneal techniques is reduced operative time and improved cosmetic results [20, 21, 67, 68]. Although both techniques close the ring, we did not include them in this analysis as we felt the approach was too different. A recent systematic review was published comparing intraperitoneal vs extraperitoneal hernia repairs [69]; however, this still included a number of different techniques (both LS and LSDS) in both groups and had no minimum follow-up period, thus making an accurate determination of recurrence rates difficult. A further review would be needed to fully address the extraperitoneal technique; however, as the basis of the operation is the same we would anticipate that without sac disruption the recurrence rate would remain higher than for an LSDS repair.

Although there is a 50% reduction in recurrence rate between the two techniques this only equates to an actual reduction of 0.7% as the overall recurrence rate remains low for both techniques. The LS technique has tens of reported modifications and has widely reported low recurrence rates. However, this is a high volume procedure, in England alone in 2019, 6491 children had an inguinal hernia repair. Therefore, if a technique like the LSDS that is based on recreating the open technique is to be adopted as the procedure of choice for laparoscopic hernia repairs, then hundreds of children could potentially avoid redo operations worldwide each year.

Setting standards and comparing outcomes between individual centers is always difficult in pediatric surgical practice, given that the volume of individual cases is often low and this is compounded further when complication rates are low. However, this study identified an overall recurrence rate of < 1% for over 6000 LSDS hernia repairs. We suggest that this should be the outcome towards which surgeons should be aiming for in their practice and one that other techniques should be tested against when conducting large studies.

## Conclusion

The LSDS technique for the repair of pediatric inguinal hernias is a safe technique which has the benefits of a laparoscopic repair while replicating the steps of the open operation. This review identified a significant reduction in recurrence rate when it was compared to the LS technique, but the studies included -especially RCTs—had moderate to high risk of bias. Large-scale multicentric prospective RCTs are needed for good quality results and to decrease confounding factors.

### Supplementary Information

Below is the link to the electronic supplementary material.

Supplementary file 1 (DOCX 13 KB)—Table 1. Combined MINORs score for comparative studies (Maximum score 24 points).

Supplementary file 2 (DOCX 13 KB)—Table 2. Combined MINORs score for non-comparative studies (Maximum score 16 points).

Supplementary file 3 (DOCX 13 KB)—Table 3. Combined RoB-2 score for Randomized controlled trials.

## Data Availability

All study data is available on request.
