# Supplementary material for: Laparoscopic hernia repair in children: does recreating the open operation improve outcomes? A systematic review
Source: Hernia. 2023 Mar 23;27(5):1037–46. doi: 10.1007/s10029-023-02772-5 (PMC10533621; doi:10.1007/s10029-023-02772-5)
Supplement: Supplementary file 1 — (DOCX 13 KB)—Table 1. Combined MINORs score for comparative studies (Maximum score 24 points). [file 10029_2023_2772_MOESM1_ESM.docx]

**Table S1. Combined MINORs score for comparative studies** (Maximum score 24 points)

| **Combined MINORs score for comparative studies (Maximum score 24 points)** | |
| --- | --- |
| **Reference** | **Combined MINORs score** |
| Al metaher HA | 18 |
| Lee SR | 18 |
| Shalaby R | 13 |
| Tsai YC | 17 |
| Lin CD | 16 |
| Shehata SM | 13 |
| Ho IG | 18 |
| Koivusalo AI | 17 |
| Lee SR | 16 |
| Marta A | 16 |
| Mishra PK | 14 |
| Nah SA | 16 |
| Saranga-Bharathi R | 17 |
| Schier F | 13 |
| Shalaby R | 15 |
| Steven M | 16 |
| Walsh CM | 17 |
| Yildiz A | 19 |
| Wang F | 17 |
| Shou T | 20 |
| Korkmaz M | 16 |
| Kozlov Y | 16 |
| Karadag CA | 14 |
