# Supplementary material for: Laparoscopic hernia repair in children: does recreating the open operation improve outcomes? A systematic review
Source: Hernia. 2023 Mar 23;27(5):1037–46. doi: 10.1007/s10029-023-02772-5 (PMC10533621; doi:10.1007/s10029-023-02772-5)
Supplement: Supplementary file 2 — (DOCX 13 KB)—Table 2. Combined MINORs score for non-comparative studies (Maximum score 16 points). [file 10029_2023_2772_MOESM2_ESM.docx]

**Table S2. Combined MINORs score for non-comparative studies** (Maximum score 16 points)

| **Combined MINORs score for non-comparative studies (Maximum score 16 points)** | |
| --- | --- |
| **Reference** | **Combined MINORs score** |
| Becmeur F | 10 |
| Boo YJ | 12 |
| Esposito C | 7 |
| Esposito C | 7 |
| Esposito C | 10 |
| Esposito C | 10 |
| Giseke S | 8 |
| Lee SR | 9 |
| Montupet P | 10 |
| Van Batavia JP | 8 |
| Wheeler AA | 7 |
| Esposito C | 8 |
| Shehata SM | 7 |
| Chan IHY | 7 |
| Chinnaswamy P | 5 |
| Geiger S | 9 |
| Gorsler CM | 7 |
| Lee DY | 9 |
| Li S | 11 |
| Montupet P | 7 |
| Parelkar SV | 9 |
| Pastore V | 9 |
| Schier F | 9 |
| Schier F | 7 |
| Schier F | 5 |
| Shalaby R | 9 |
| Turial S | 9 |
| Turial S | 11 |
| Ur RF | 8 |
