# Supplementary material for: Laparoscopic hernia repair in children: does recreating the open operation improve outcomes? A systematic review
Source: Hernia. 2023 Mar 23;27(5):1037–46. doi: 10.1007/s10029-023-02772-5 (PMC10533621; doi:10.1007/s10029-023-02772-5)
Supplement: Supplementary file 3 — (DOCX 13 KB)—Table 3. Combined RoB-2 score for Randomized controlled trials. [file 10029_2023_2772_MOESM3_ESM.docx]

**Table S3. Combined RoB-2 score for Randomized controlled trials**

| **Combined RoB-2 score for Randomized controlled trials** | |
| --- | --- |
| **Reference** | **Combined RoB-2 score** |
| Koivusalo AI | Some concerns |
| Shalaby R | High risk of bias |
| Abd-Alrazek M | Some concerns |
| Celebi S | High risk of bias |
| Hasanein A | Some concerns |
| Pant N | High risk of bias |
| Albatarny AM | High risk of bias |
| Borkar NB | High risk of bias |
